# Raloxifene injections normalize age-related mechanical sensitization in female and male mice and augment intervertebral disc structure in old female mice

**DOI:** 10.1016/j.joca.2026.03.118

**Published:** 2026-03-19

**Authors:** Neharika Bhadouria, Jonathan J. Huang, Janai Augustin, Bowen Wang, Joana I. Almeida, Tori Kroon, James Manolis, Michael Lemonick, Alan Seifert, Deepak Vashishth, James C. Iatridis, Nilsson Holguin

**Affiliations:** a Dept. of Orthopedics, Icahn School of Medicine at Mount Sinai, New York, NY, United States; b Ctr. for Biotech. & Interdisciplinary Studies, Rensselaer Polytechnic Institute, New York, NY, United States; c Ctr. for Eng. & Precision Medicine, Rensselaer-Icahn School of Medicine at Mount Sinai, New York, NY, United States; d Biomedical Engineering and Imaging Institute, Dept. of Radiology, Icahn School of Medicine at Mount Sinai, New York, NY, United States

**Keywords:** Hormone Replacement/Receptor Modulators, Aging, Therapeutics, Spine

## Abstract

**Objective:**

Intervertebral disc (IVD) degeneration (IVDD) is a prevalent contributor to low back pain, yet IVDD-modifying therapeutics are unavailable. Osteoporosis drug raloxifene reduces age- and sex-related IVDD and indications of pain in preclinical and early-stage clinical studies, but the impact of raloxifene on evoked and spontaneous pain-related behavior is unclear. This study evaluated the effects of raloxifene injections on IVD structure and function, and pain-related behavior in 4-month-old and 24-month-old, female and male mice.

**Design:**

Raloxifene was injected to 4-month-old and 24-month-old, female and male mice for 4 weeks, 5×/week or 4 days. Mice were assayed for pain by mechanical sensitivity and gait dynamics, neurotransmitter substance P expression, and IVD biomechanical function, function and hydration.

**Results:**

Raloxifene injections reduced mechanical sensitivity and IVD substance P in 4-month-old and 24-month-old mice of both sexes with age and sex-differences measured for gait dynamics and IVD morphology. Raloxifene reduced IVDD in 4-month-old and 24-month-old female mice (4-month-old: −44%, p=0.03;24-month-old: −32%, p=0.02), but did not affect male IVDD grade (4-month-old: +7%, p=0.35; 24-month-old: −14%, p=0.34). Similarly, raloxifene increased propel-stance duration in 24-month-old female mice by 4% (p=0.01), but did not affect gait in 24-month-old male mice(−0.4%, p=0.94). Using microMRI, 4 days of raloxifene injections increased IVD hydration from baseline by 4% (p=0.046).

**Conclusions:**

Raloxifene injections significantly reduced IVDD in female mice and reduced mechanical sensitivity to evoked mechanical pain in both male and female mice, suggesting that raloxifene may serve as a therapeutic for painful age-related IVDD.

## Introduction

Intervertebral disc (IVD) degeneration (IVDD) is a leading cause of low back pain and physical disability [1], with costs exceeding $100 billion in the United States of America [2]. Healthy IVDs transmit complex mechanical loading along the spine, while enabling spinal flexibility. Natural IVD aging is comprised of extracellular matrix breakdown by loss of hydrophilic proteoglycans, leading to IVD height loss and mild biomechanical dysfunction [3–6]. Aging exacerbates IVDD, which can cause pain by excessive IVD deformation and instability [7–9], mechanical failure in the form of fissures/tears [10], sensitization from neo-innervation and inflammation [11–13], and bone-on-bone contact from severe IVD collapse [14]. Tissue-level degeneration disrupts the cellular microenvironment [15] and promotes cell maturation, senescence and apoptosis, which instigates a vicious cycle of IVDD by impairing extracellular matrix synthesis [16]. Currently, there are no IVDD-disease modifying therapeutics that reduce discogenic back pain by augmentation of IVD structure and hydration or retardation of IVDD progression.

Raloxifene hydrochloride is a Food and Drug Administration-approved selective estrogen receptor modulator prescribed to postmenopausal women to treat and prevent osteoporosis and to reduce the risk of vertebral fracture, which may also promote IVD anabolism and function. In bone, raloxifene binds estrogen receptors (ER) on osteoclasts to inhibit bone resorption [17] and, independent of cells, biophysically binds water to collagen [18,19]. In clinical studies, raloxifene increases IVD height [20] and relieves self-reported back pain in pre- and post-menopausal women [21]. Similarly, in a preclinical study [22], raloxifene injections to young-adult and old female mice increase IVD height, promote extracellular matrix anabolism, and stiffen IVD mechanical properties. Therefore, raloxifene holds potential to be repurposed for discogenic pain by augmentation of IVD structure and function in old postmenopausal women, where developing effective, non-surgical treatments are a priority [23].

Separately, raloxifene injections may regulate pain intensity independent of the structural benefits to the IVD [24]. Injections of raloxifene to young-adult female and male mice suppress IVD expression of neurotransmitter substance P (neurokinin-1) [22] despite lack of benefits to IVD structure and function in young-adult male mice. Substance P is nerve signaling neurotransmitter and nociceptive pain marker that is associated with painful IVDD [25] and is negatively associated with estrogen receptor-α (ER-α) protein expression in the IVD of postmenopausal women [26]. Signaling of raloxifene through ER-α may reduce nerve signaling within and beyond the IVD.

The IVD is lowly vascularized [27], but increasing the raloxifene dosage to enhance efficacy for IVDD-related conditions must balance the adverse sequelae of raloxifene treatments at high dosages. Postmenopausal women are prescribed oral administration of 60 mg of raloxifene per day (∼1 mg/kg/d), where it is quickly and widely absorbed [28] with few reported side effects. However, raloxifene increases the incidence of thromboembolic events to 1% of treated women compared to 0.3% in placebo-treated women [29]. Corroboratively, factors associated with pro-thromboembolism increase with dosage of raloxifene [30]. Therefore, the effect of raloxifene dosage on IVD structure and function must be studied further.

Here, we proposed a mouse study to investigate the effect of raloxifene on IVD structure/function and pain-related behavior in two aims: i) determine the effect of injections of raloxifene on IVD structure, IVD biomechanics, and pain-related behavior in young-adult and old, female and male mice, and ii) determine whether low doses of raloxifene injections can improve IVD structure, function and hydration due to its ability to biophysically bind water to collagen, since low-dose raloxifene is likely to have little stimulation of estrogen signaling. We hypothesized that i) tail vein injections of raloxifene injections would augment IVD structure and function and reduce pain-related behavior in young-adult and old, female and male mice, and ii) low dosages of raloxifene injections would augment IVD structure, function and hydration in young-adult female mice.

## Materials and methods

### Mice

All mice were housed in the institutional animal facility under a 12-hour light/dark cycle, with access to standard chow and water *ad libitum*. The mice were randomly assigned for injections to ensure that the average mouse weight was balanced across all groups prior to administering any injections. First, female and male C57BL/6J mice (n=3–5 per group) were tail vein injected with 0.5 mg/kg of raloxifene hydrochloride (Tocris Bioscience: Cat#82640–04–8, RAL) or with PBS (vehicle, VEH) five times per week for 4 weeks, beginning at either 2.5 or 22.5 months of age. Data was collected at 4 or 24 months of age post-injection. We changed the delivery method of raloxifene injections from subcutaneous injections [22] to tail vein injections because we intend to use a coccygeal IVD degeneration model, but subcutaneous injections of raloxifene do not augment coccygeal vertebral bone structure nor coccygeal IVD in female mice likely due to limited bioavailability (Supplemental Figure 1, Supplemental Table 1). A power analysis (α=0.05, β=0.80, p < 0.05) of ER-α protein expression in annulus fibrosus cells between young-adult female mice treated with vehicle or raloxifene from our previous published article [22] suggest n=4 per group. Next, 4-month-old female C57BL/6J (n=3–5 per group) mice received tail vein injections of 0.5 mg/kg of RAL or VEH for 4 consecutive days. Animals were assessed for pain tolerance using hind paw mechanical sensitivity and gait. After the last injection of raloxifene and euthanasia by administration of carbon dioxide at a flow rate of 30–70% volume per minute, coccygeal motion segments (vertebra-IVD-vertebra) were analyzed for outputs related to IVD and vertebral bone structure, composition, and function (Supplemental Table 2). The sample sizes and exclusion criteria for all main and supplemental figures are provided in Supplemental Tables 3 and 4.

### Behavioral assays

#### Evoked pressure tolerance: measurement of mechanical stimuli-induced pain

Hind paw mechanical sensitivity was evaluated using calibrated von Frey monofilaments (Stoelting Co.) and quantified by integration of the ‘percent response’ across filament force[31], which represents the sensitivity the paw mechanical stimulation. Prior to assessment, mice were acclimated to handling and the testing environment for 30 min on the day of testing. The maximum paw withdrawal threshold was defined as the lowest filament force that elicited a nocifensive response in at least 60% of trials (3 out of 5 applications). The response to each filament force (0.2–15 g) is graphed and area under curve (AUC) is calculated as the area for each animal until the 60% response [32] (Method Supplemental 1).

#### Spontaneous pain: assessment of gait kinematics

We performed gait analysis using the DigiGait™ Imaging System (Mouse Specifics Inc., Boston, MA), which utilizes Ventral Plane Imaging Technology to capture digital paw prints as mice walked on a motorized treadmill belt [32]. Using DigiGait™ software (Mouse Specifics Inc.), we analyzed gait parameters, including %brake stance, %propel stance, among others [32,33]. All assessments were performed in a blinded manner to minimize bias (Method Supplemental 2).

### Histology and Immunohistochemistry

IVDD score, IVD height and disc height index were assessed from IVD stained with Safranin-O/Fast Green (Saf-O/FG) (Method Supplemental 3). The IVDD score was determined by evaluating the annulus fibrosus (AF), nucleus pulposus (NP), endplate (EP) and the AF-NP boundary, with a total score ranging from 0 (normal, healthy IVD) to 14 (severe degeneration) [34]. To investigate the role of estrogen signaling, we performed staining for estrogen receptor-α (ER-α). Additionally, to assess pain-related signaling, we stained for the neuropeptide substance P (Method Supplemental 3).

### Fourier transform infrared (FTIR) spectroscopy and analysis

The fourier transform infrared (FTIR) spectra were acquired from a Bruker Vertex 70 FTIR Spectrometer (Bruker Scientific LLC, Billerica, MA, USA) using attenuated total reflectance mode as previously described [35]. Following pad-drying with Kimwipes and air-drying under room temperature for one hour to remove superficial water, 256 scans were obtained from caudal coccygeal IVD CC12–13 with a spectral resolution of 2 cm^−1^. Peak identification, deconvolution, and integration were conducted in OriginPro software (version 2025, OriginLab Corp., Northampton, MA, USA). FTIR spectral properties of the IVDs, including bound water/amide I, proteoglycan/amide I, collagen integrity, amide II/amide I, and collagen enzymatic cross-linking, were subsequently quantified (Method Supplemental 4).

### Biomechanical analysis

Axial tension-compression and torsional tests were performed on CC6–7 motion segments (vertebra-IVD-vertebra) using an Electroforce 3200 and an AR2000-EX rheometer (TA Instruments, New Castle, USA) under force- and angular displacement-controlled protocols, respectively. Axial and torsional properties were calculated from the 19th cycle using MATLAB (MathWorks, Inc., Natick, USA). Axial tension-compression properties included compressive stiffness, tensile stiffness, range of motion (ROM), neutral zone (NZ) length, NZ stiffness and hysteresis. Torsional properties included torque range, stiffness and energy dissipation (hysteresis) **(**Method Supplemental 5**)**.

### Micro-computed tomography (μCT)

Motion segments CC6–7 were harvested and maintained in 1xPBS until ready for μCT scanning. CC6 was imaged using the Scanco40 μCT (SCANCO Medical AG) at a resolution of 9.9 μm. Contouring and analysis of trabecular and cortical bone followed the protocol established in our previous studies [22]. Trabecular bone parameters included bone volume fraction (Tb.BV/TV), trabecular thickness (Tb.Th), trabecular number (Tb.N), and volumetric bone mineral density (vBMD). Cortical bone parameters assessed included cross-sectional thickness (Ct.Th), tissue mineral density (TMD), area (Ct.Ar), medullary area (Ma.Ar), tissue area (Tt.Ar) and minimum moment of inertia (I_min_). A lower threshold of 262 (582.7 mg HA/ccm) and the upper threshold of 1000 (2778.9 mg HA/ccm) were applied for analysis.

### qPCR

Gene expression of the extracellular matrix marker *aggrecan* and the estrogen signaling marker, *ER-α* was evaluated in VEH and RAL groups from the 4-day injection study using 4-month-old female IVDs. (Method Supplemental 6).

### microMRI

Animals were anesthetized using Isoflurane (1–1.5%) and positioned vertically (head up) in the scanner bore such that the caudal coccygeal CC7–8 vertebra in the isocenter of the magnet. The tail position was restrained by a small tube to help with MR image planning. Respiration rate was monitored using a pillow-sensor (SAInsturments, Stony Brook, NY). MRI data were acquired on a bruker 9.4 T scanner (Bruker BioSpin GmbH, Ettlingen, Germany) equipped with AvanceIIIHD console and a 30 mm head coil (RAPID Biomedical GmbH, Rimpar, Germany) running ParaVision6.0 software. The multi-echo spin-echo was fitted to a single exponential decay to map T2, and then T2 was the marker of hydration (Method Supplemental 7).

### Statistical analysis

Statistical analyses were performed using GraphPad Prism (V10.3.1). Data are presented as group mean/standard deviation for significant or trending comparisons. Unpaired Student’s T-tests compared the effect of aging (VEH of 4-month-old vs 24-month-old) and raloxifene injections (VEH vs RAL). Paired Student’s T-Test compared longitudinal data (pre- versus post-injection). Multiple pairwise comparisons were conducted for each experiment, and no formal correction for multiple comparisons was applied because the goal of the study is to determine the impact of raloxifene injections on IVD morphology and pain-related behavior in each sex and age rather than between each. The significance threshold for all statistical analyses was p < 0.05.

## Results

### Raloxifene injections for 4 weeks normalized the age-related mechanical sensitization of old female and male mice, and shifted the percentage of gait stance towards propulsion in old female mice and not in male mice

**Fig. 1:**
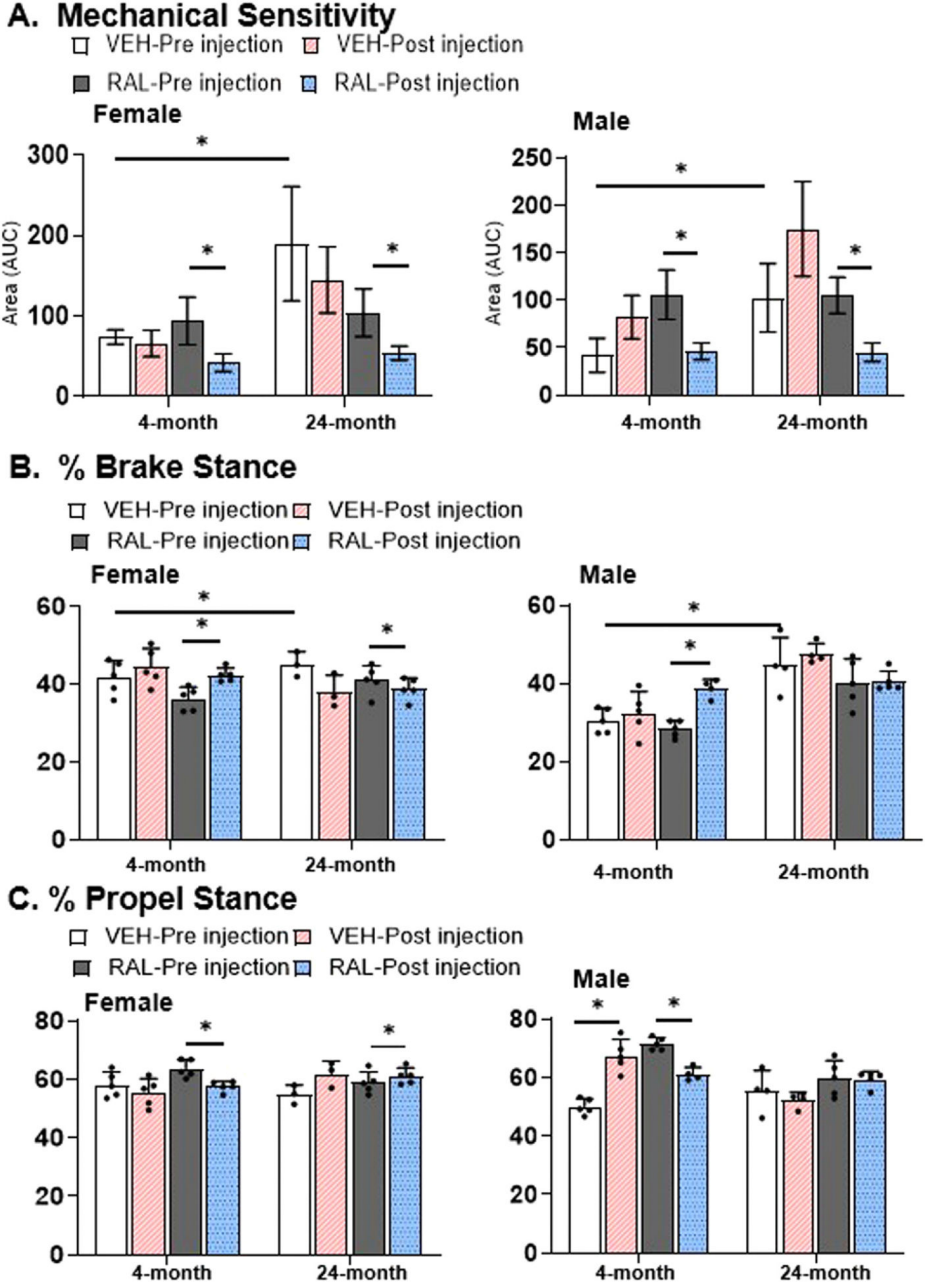
Raloxifene injections for 4 weeks reduced mechanical sensitivity in old female and male, and shifted the percentage of gait stance towards propulsion in old female mice and not in male mice. (A) Mechanical sensitivity by von Frey as the area under the curve at 60% response and spontaneous pain measured by gait kinematics of (B) % brake stance and (C) % propel stance of 4-month and 24-month-old, female (left side) and male (right side) mice injected with vehicle or raloxifene. Unpaired Student’s T-tests compared the effect of aging (VEH of 4-month-old vs 24-month-old) and raloxifene injections (VEH vs RAL). Paired Student’s T-Test compared longitudinal data (pre- versus post-injection). The significance threshold for all statistical analyses was *p < 0.05. VEH-Vehicle group injected with PBS, RAL-Raloxifene injected group.

Aging increased the sensitization to mechanical stimuli as noted by greater response-filament force area in female and male mice by 156%(4-month-old:74 /8.9, 24-month-old:190/71.2) and 144% (4-month-old:42 /17.8, 24-month-old:102.5/35.94), respectively (Fig. 1A, Supplemental Figure 2). Compared to pre-injection, vehicle injections did not change mechanical sensitivity in 4-month-old or 24-month-old, female or male mice. By contrast, raloxifene injections decreased mechanical sensitivity in female 4-month-old and 24-month-old mice by 55% [RAL-Pre injection: 94/29.7, RAL-Post injection: 42/11.0] and 48% [RAL-Pre injection: 104/29.7, RAL-Post injection: 54/8.9], respectively, and male mice responded similarly [−56%, 4-month-old: RAL-Pre injection:106/26.0, RAL-Post injection: 46/8.9; −57%, 24-month-old: RAL-Pre injection:105/19.0, RAL-Post injection: 45/10.0].

In gait kinematics, aging increased % brake stance in female and male mice by 8% [4-month-old: 41.8/4.3, 24-month-old: 45.1/3.2] and 46% [4-month-old: 30.5/3.2, 24-month-old: 44.7/7.1], respectively, indicating reduced deceleration control, without altering the percentage of propel stance in either female or male mice (Fig. 1C, D). Vehicle injections did not alter % brake stance in 4- or 24-month-old, female and male mice (Fig. 1B). By contrast, raloxifene injections increased the % brake stance in 4-month-old female mice by 17% [RAL-Pre injection:36.3/3, RAL-Post injection:42.4/1.8] and decreased % brake stance in 24-month-old females by 5% [RAL-Pre injection:41.1/3.7, RAL-Post injection:38.9/2.7], suggesting an age-dependent modulation of locomotor dynamics in response to injection of raloxifene (Fig. 1B). In 4-month-old males, raloxifene injections increased % brake stance by 37% [RAL-pre injection:28.4/2.1, RAL-post injection:38.7/2.3], but raloxifene injections did not alter it in 24-month-old male mice. Similarly, raloxifene injections decreased % propel stance in 4-month-old female mice by 10% [RAL-Pre injection:63.7/3.0, RAL-Post injection:57.6/1.8], and increased it in 24-month-old female mice by 4% [RAL-Pre injection:58.9/3.7, RAL-Post injection:61.1/2.7] without changes induced by vehicle injections (Fig. 1C). By contrast, raloxifene injections attenuated the vehicle injection-induced % propel stance increase of 37% [VEH-Pre injection:50.2/2.7, VEH-Post injection:67.6/5.5] by 14% [RAL-Pre injection:71.6/2.1, RAL-Post injection:61.3/2.3] in 4-month-old male mice, but % propel stance in 24-month-old male mice was not impacted by injections of vehicle or raloxifene (RAL-Pre injection: 59.8/6.1, RAL-Post injection: 61.3/2.3; Fig. 1C). Other gait kinematic outcomes indicated that vehicle injections influenced mouse behavior more so than raloxifene injections (Supplemental Table 5).

### Raloxifene injections for 4 weeks reduced the fraction of protein substance P-expressing IVD cells in young-adult and old, female and male mice

**Fig. 2:**
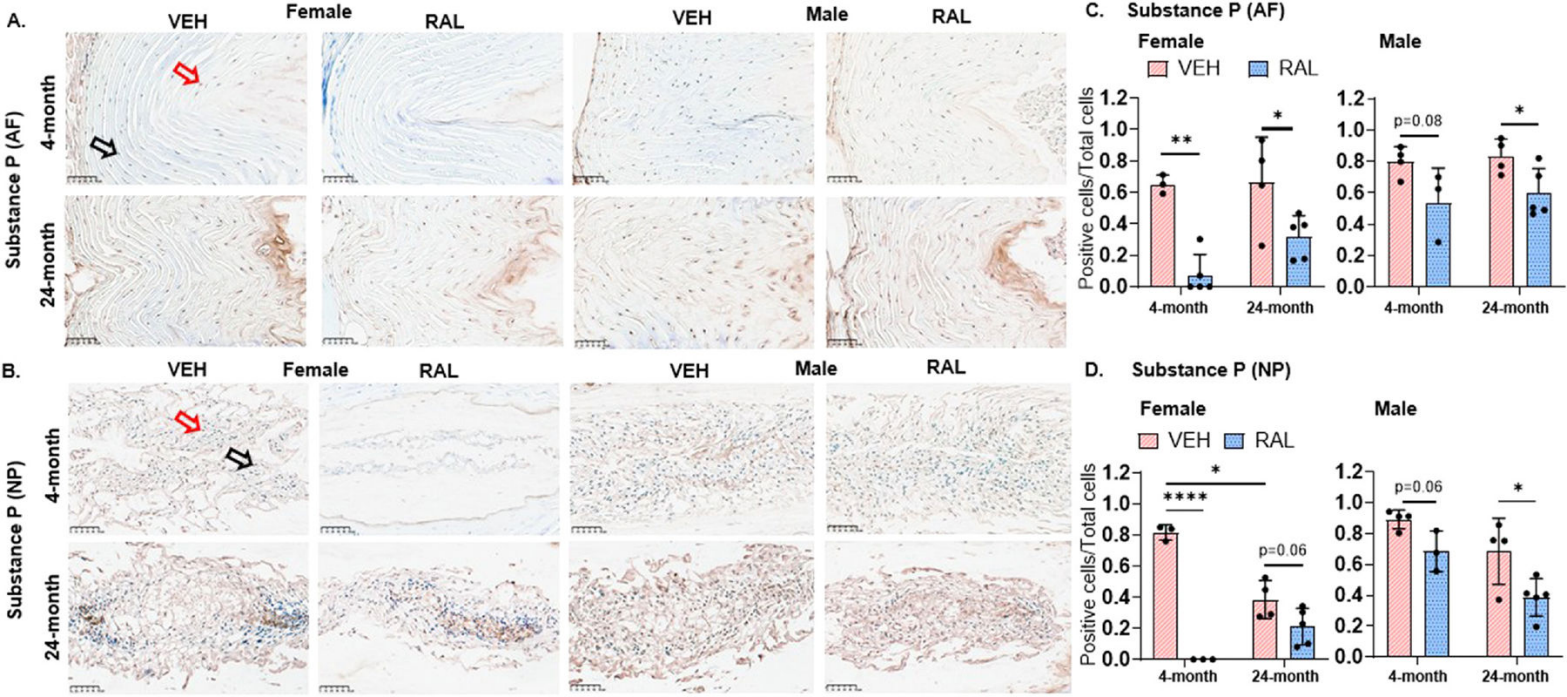
Raloxifene injections for 4 weeks decreased the fraction of protein substance P-expressing cells in young-adult and old, female and male mice. (A) Representative 40× immunohistochemistry-stained images of SubP (substance P) expression in the annulus fibrosus (AF) and nucleus pulposus (NP) of 4- and 24-month-old female (left side) and male (right side) mice. Quantification of the ratio of substance P-expressing cells to total cells within the (B) AF and (C) NP. Red arrows indicate positive staining for substance P, whereas black arrows indicate absence of staining for substance P. Unpaired Student’s T-tests compared the effect of aging (VEH of 4-month-old vs 24-month-old) and raloxifene injections (VEH vs RAL). The significance threshold for all statistical analyses was *p < 0.05. VEH-Vehicle group injected with PBS, RAL-Raloxifene injected group.

Aging decreased the fraction of substance P-expressing nucleus pulposus (NP) cells in female IVDs by 52% [4-month-old:0.82/0.05, 24-month-old:0.38/0.12], but aging did not affect the same in males or of substance P-positive annulus fibrosus (AF) cells in female or male mice (Fig. 2A-D). In 4- and 24-month-old AF cells, raloxifene injections reduced the fraction of substance P-expressing cells by 88% [VEH:0.65/0.06, RAL:0.66/0.29] and 52% [VEH:0.66/0.29, RAL:0.32/0.14] in female mice, respectively (Fig. 2A,C), and by a trending 32% [VEH:0.80/0.09, RAL:0.54/0.22, p=0.08] and 28% [VEH:0.83/0.11, RAL:0.60/0.16] in male mice, respectively (Fig. 2A,C). The reduction of substance P by raloxifene injections was similar in NP cells, except for a trending (23%, VEH:0.38/0.12, RAL:−0.21/0.12, p=0.06) reduction by raloxifene injections in 24-month-old female mice (Fig. 2D).

### Raloxifene injections for 4 weeks increased the fraction of protein ER-α-expressing IVD cells in young-adult and old, female and male mice

**Fig. 3:**
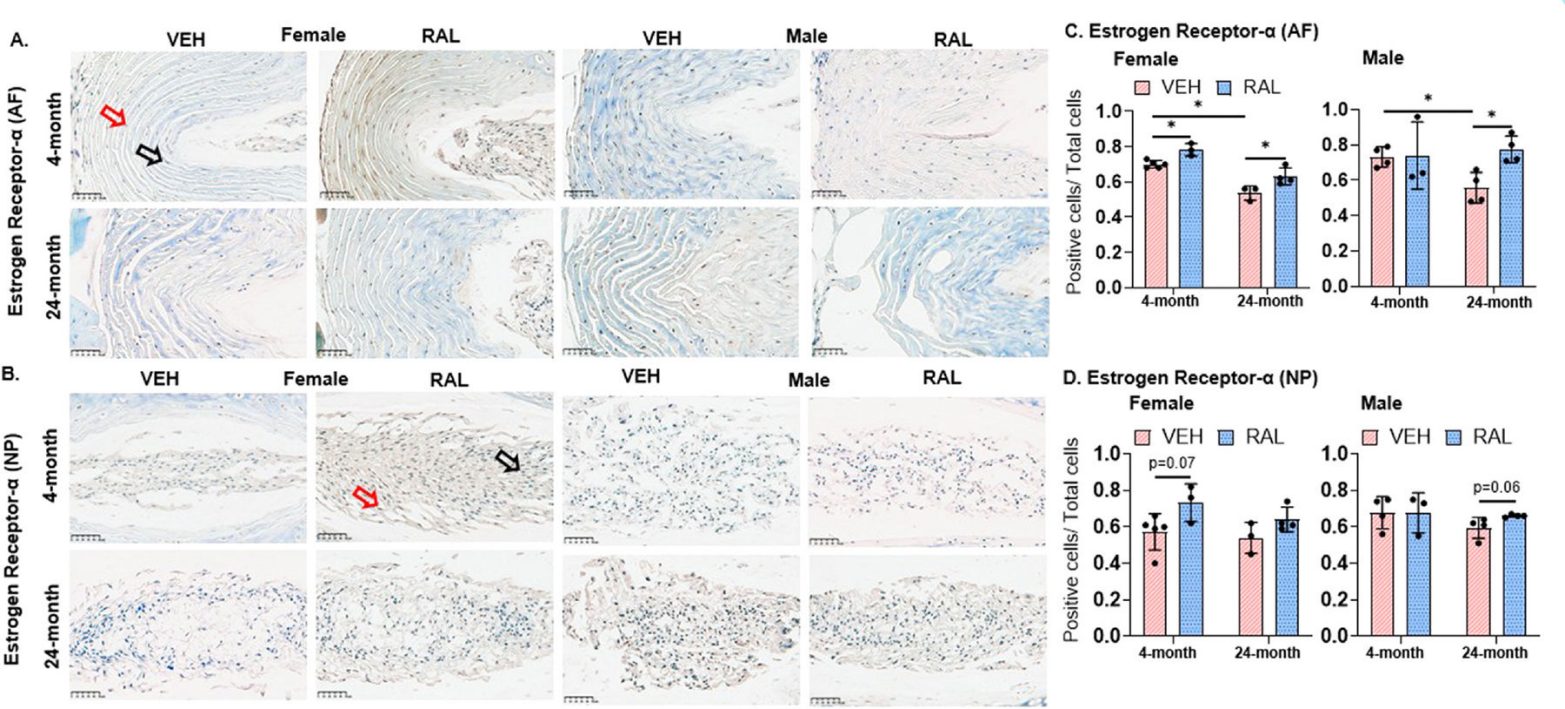
Raloxifene injections for 4 weeks increased the number of protein estrogen receptor -α-expressing IVD cells in young-adult and old, female and male mice. (A) Representative 40× immunohistochemistry-stained images of protein estrogen receptor- α (ER-α) expression in the annulus fibrosus (AF) and nucleus pulposus (NP) of 4- and 24-month-old female (left side) and male (right side) mice. Quantification of the ratio of ER-α positive cells to total cells within the (B) AF and (C) NP. Red arrows indicate positive staining for ER-α, whereas black arrows indicate absence of staining for ER-α. Unpaired Student’s T-tests compared the effect of aging (VEH of 4-month-old vs 24-month-old) and raloxifene injections (VEH vs RAL). The significance threshold for all statistical analyses was *p < 0.05. VEH-Vehicle group injected with PBS, RAL-Raloxifene injected group.

Aging reduced the fraction of ER-α-positive AF cells in female and male IVDs by 23% [4-month-old: 0.70/0.02, 24-month-old:0.54/0.04] and 24% [4-month-old:0.73/0.06, 24-month-old:0.56/0.09], respectively, but aging did not alter the fraction of ER-α-expressing NP cells in mice of either sex (Fig. 3A-D). In AF cells, raloxifene injections increased the fraction of ER-α-expressing cells in 4- and 24-month-old female mice by 12% [VEH:0.70/0.02, RAL:0.78/0.04] and 18% [VEH:0.54/0.04, RAL:0.63/0.05] respectively (Fig. 3A,C). By contrast, raloxifene injections increased ER-α-positive cells in 24-month-old male mice by 42% [VEH: 0.56/0.09, RAL:0.78/0.08], but raloxifene did not alter the same in 4-month-old male mice (Fig. 3C). In NP cells, raloxifene injections did not alter the fraction of ER-α-positive cells but trended to increase it in 4-month-old female mice (21%, VEH:0.57/0.10, RAL:0.73/0.10) and 24-month-old male mice (10%, VEH:0.54/0.09, RAL:0.64/0.07, p=0.06, Fig. 3B, D).

### Raloxifene injections for 4 weeks increased IVD expression of extracellular matrix constituents and bound water by FTIR in female mice and not in male mice

**Fig. 4:**
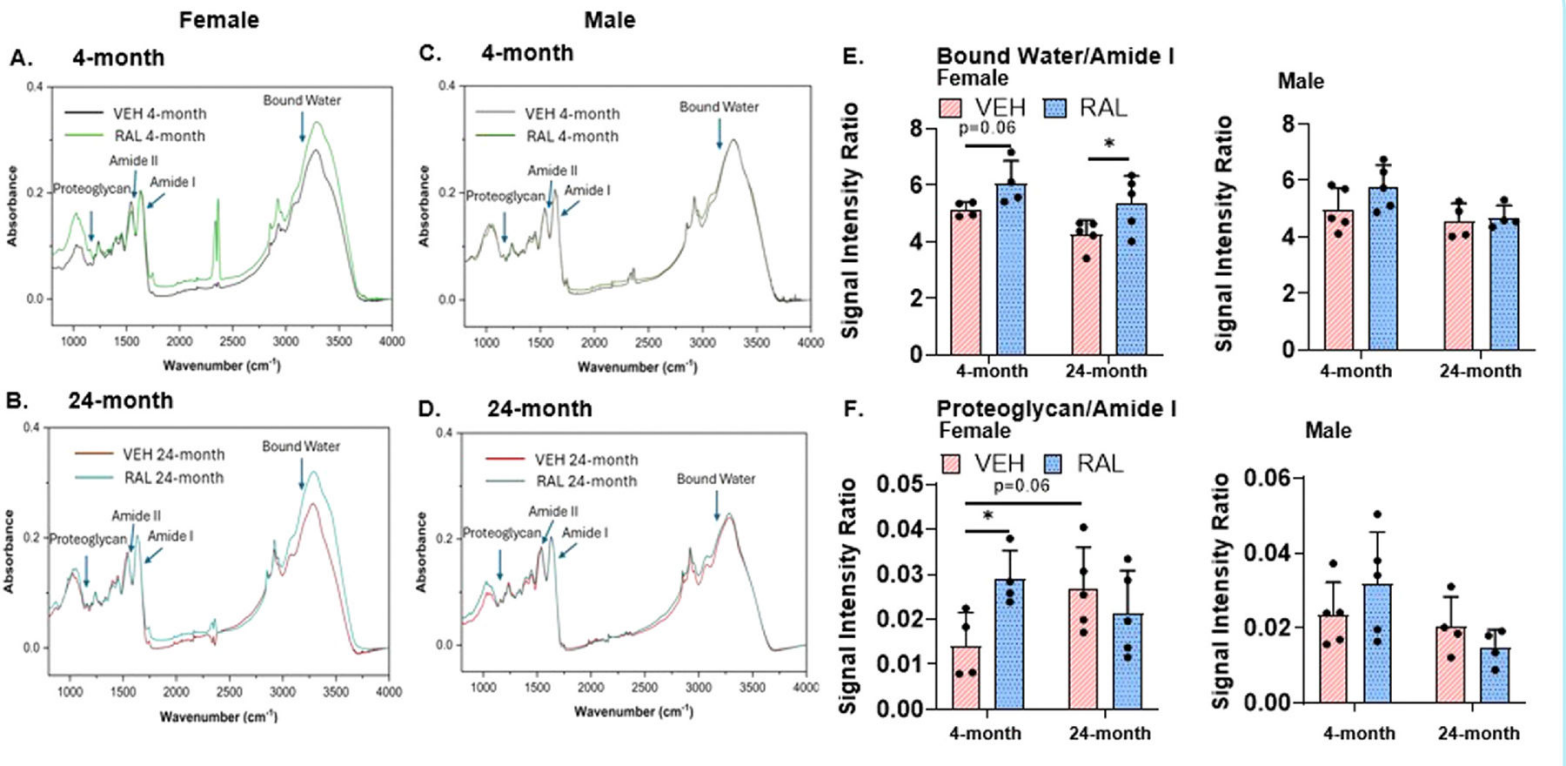
Raloxifene injections for 4 weeks sex-dependently increased IVD expression of extracellular matrix constituents by FTIR. Representative FTIR spectra comparing VEH and RAL-treated groups for IVDs from (A) 4-month-old female mice (left side), (B) 24-month-old female mice (left side), (C) 4-month-old male mice (right side), and (D) 24-month-old male mice (right side). Quantitative analysis of signal intensity ratios for (E) bound water/amide I and (F) proteoglycan/amide I of caudal coccygeal IVD. Unpaired Student’s T-tests compared the effect of aging (VEH of 4-month-old vs 24-month-old) and raloxifene injections (VEH vs RAL). The significance threshold for all statistical analyses was *p < 0.05. VEH-Vehicle group injected with PBS, RAL-Raloxifene injected group.

In female mice, aging did not affect IVD bound water/amide I ratio, but aging trended to increase IVD proteoglycan/amide I ratio (87%, 4-month-old:0.01/0.01, 24-month-old:0.03/0.01, p=0.06; Fig. 4A-F). In females, raloxifene injections trended to increase bound water/amide I ratio in 4-month-old IVDs (17%, VEH:5.15/0.26, RAL:6.07/0.80, p=0.06) and increased it 24-month-old IVDs by 20% (VEH:4.27/0.51, RAL:5.37/0.97; Fig. 4E). Further, in females, raloxifene injections increased the proteoglycan/amide I ratio in 4-month-old mice by 83% (VEH:0.01/0.01, RAL:0.03/0.01), but not in 24-month-old mice (Fig. 4F). Neither aging nor raloxifene injections affected IVD bound water/amide I ratio or proteoglycan/amide I ratio in male mice. TV injections of raloxifene induced minor changes in collagen-related IVD properties (Supplemental Table 6).

### Raloxifene injections for 4 weeks reduced the IVDD score in young-adult and old female mice

**Fig. 5:**
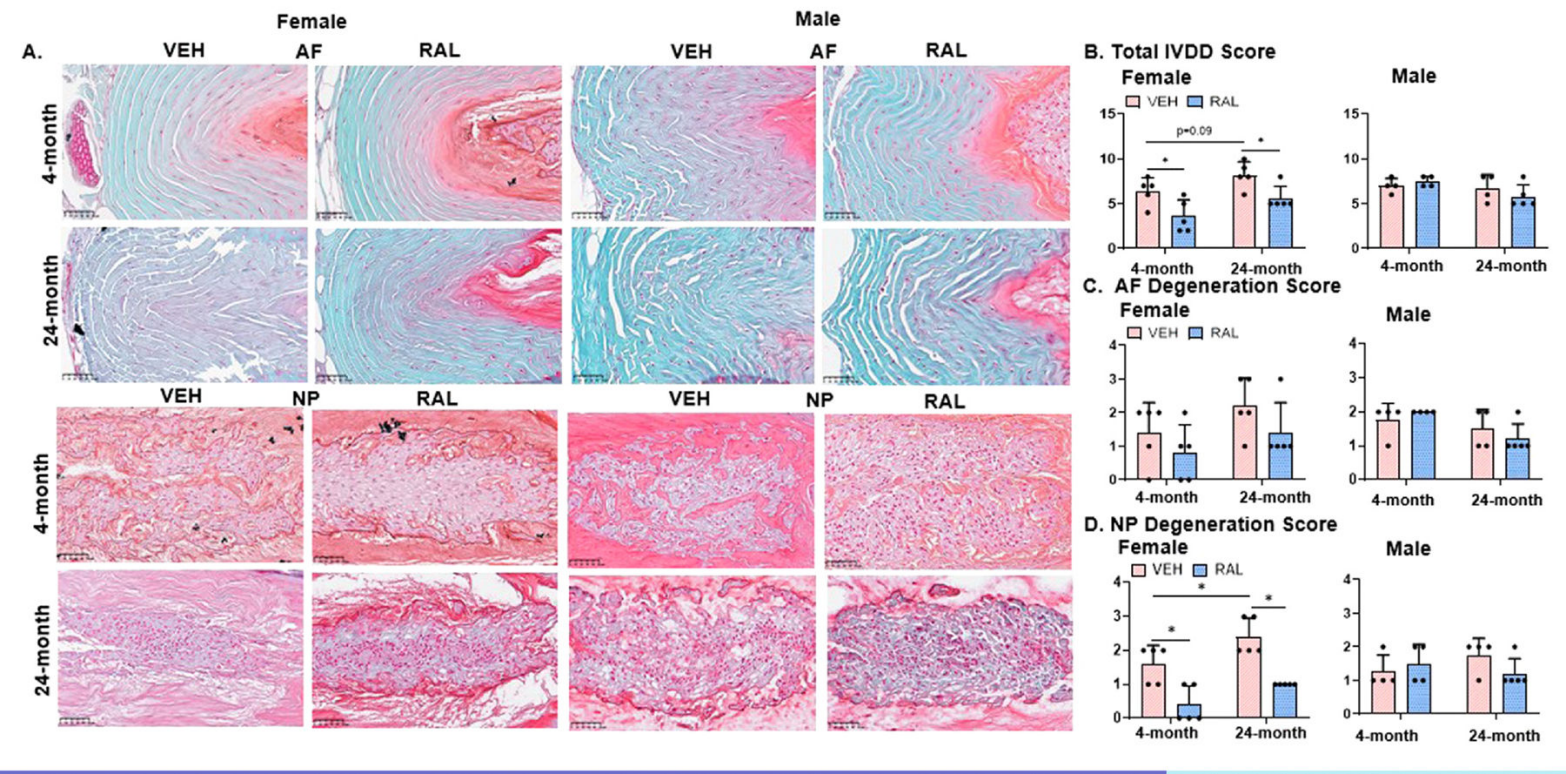
Raloxifene injections for 4 weeks reduced the IVDD score in young-adult and old female mice. (A) 20x Safranin O/Fast Green-stained representative images of IVD (AF and NP) from 4- and 24-month-old female (left side) and male (right side) mice analyzed for (B) Total IVDD score and (C) AF Degeneration Score and (D) NP Degeneration Score. Unpaired Student’s T-tests compared the effect of aging (VEH of 4-month-old vs 24-month-old) and raloxifene injections (VEH vs RAL). The significance threshold for all statistical analyses was *p < 0.05. VEH-Vehicle group injected with PBS, RAL-Raloxifene injected group. Annulus fibrosus (AF), Nucleus pulposus (NP). Scale bar: 100μm.

In female IVD, aging trended to increase the IVDD histopathological score (28%, 4-month-old:6.40/1.52, 24-month-old:8.20/1.48, p=0.09), which was driven by an increase in NP degeneration of 50% (4-month-old:1.60/0.55, 24-month-old:2.40/0.55) (Fig. 5A-E). In females, raloxifene injections reduced the IVDD score in 4- and 24-month-old IVDs by 44% (VEH:6.40/1.52, RAL:3.60/1.82) and 32% (VEH:8.20/1.48, RAL:5.60/1.34), respectively (Fig. 5A-C). Similarly, in females, raloxifene injections reduced NP degeneration scores in 4-month- and 24-month-old IVDs by 75% (VEH:1.60/0.55, RAL:0.40/0.55) and 58% (VEH:2.40/0.55, RAL:1.00/0.00) respectively, but without any changes in AF degeneration scores (Fig. 5D, E). Raloxifene injections did not alter IVDD, NP degeneration or AF degeneration in male mice (Fig. 5C-E). Neither aging nor raloxifene injections altered IVD height or disc height index, except for trending increases in IVD height of 4-month-old female (9%, VEH:329.64/13.62, RAL:348.82/17.78, p=0.09) and male mice (6%, VEH:374.06/57.65, RAL:466.90/76.50, p=0.06) with raloxifene injections (Supplemental Figure 3A, B).

### Raloxifene injections for 4 weeks stiffened axial IVD mechanical function

**Fig. 6:**
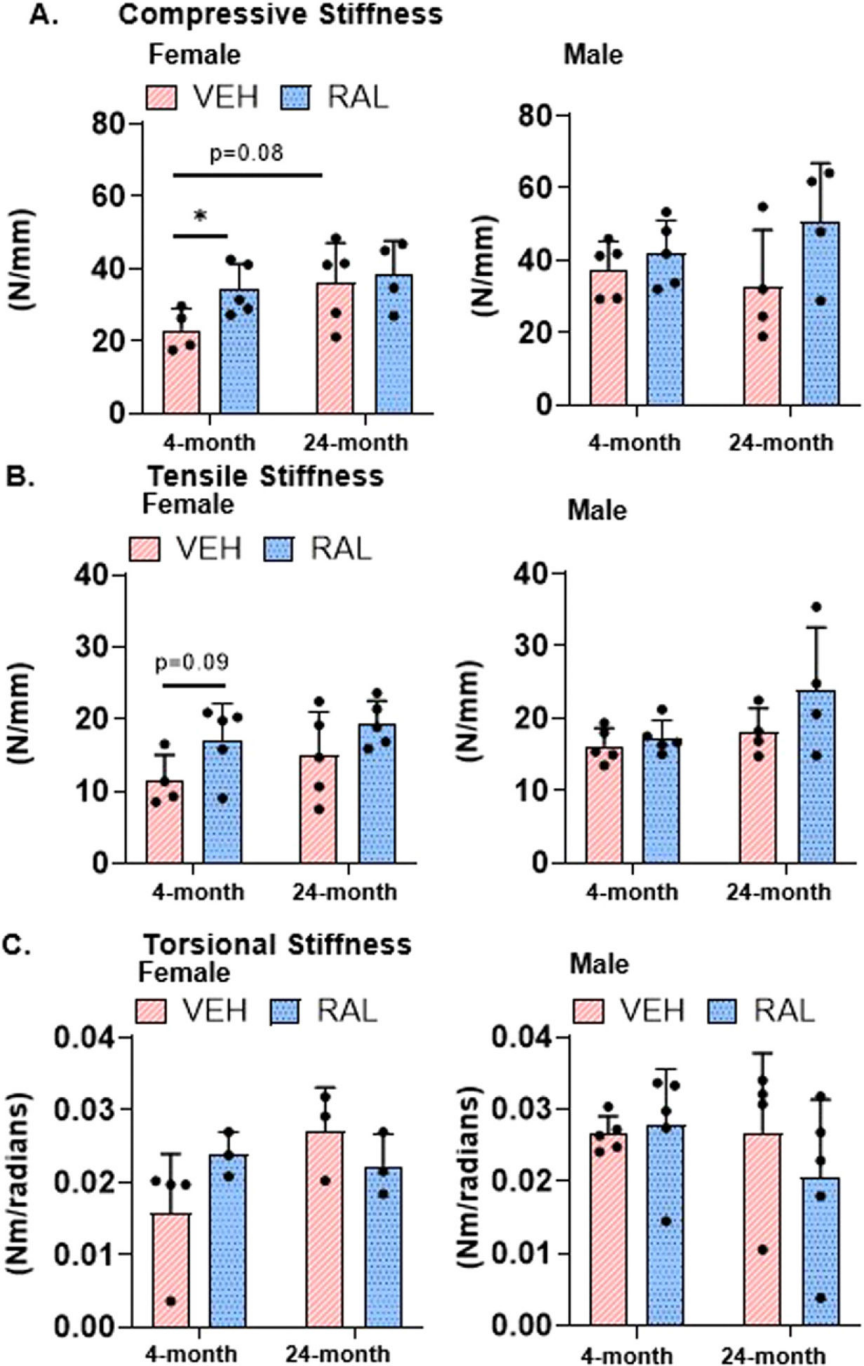
Raloxifene injections for 4 weeks stiffened axial IVD mechanical function. Axial IVD (A) compressive stiffness and (B) tensile stiffness, and torsional IVD (C) stiffness from 4-month-old and 24-month-old, female (left side) and male (right side) mice. Unpaired Student’s T-tests compared the effect of aging (VEH of 4-month-old vs 24-month-old) and raloxifene injections (VEH vs RAL). The significance threshold for all statistical analyses was *p < 0.05. VEH-Vehicle group injected with PBS, RAL-Raloxifene injected group.

In female mice, aging trended to increase axial IVD compressive stiffness (55%, 4-month-old:23.14/5.84, 24-month-old:36.01/11.16, p=0.08) and did not alter axial IVD tensile stiffness or IVD torsional stiffness (Fig. 6A-C). Raloxifene injections increased IVD compressive stiffness in 4-month-old female IVDs by 48% (VEH:23.14/5.84, RAL:34.31/7.08) and trended to increase tensile stiffness (50%, VEH:11.43/3.62; RAL:17.18/4.96, p=0.09). In male mice, neither aging nor raloxifene injections altered axial or torsional mechanical stiffness (Fig. 6A-C). No additional alterations were observed in other axial or torsional mechanical properties with aging or raloxifene injections (Supplemental Table 7).

### Short-term raloxifene injections enhanced IVD structure, function and hydration, and suppressed pain-related marker substance P

**Fig. 7:**
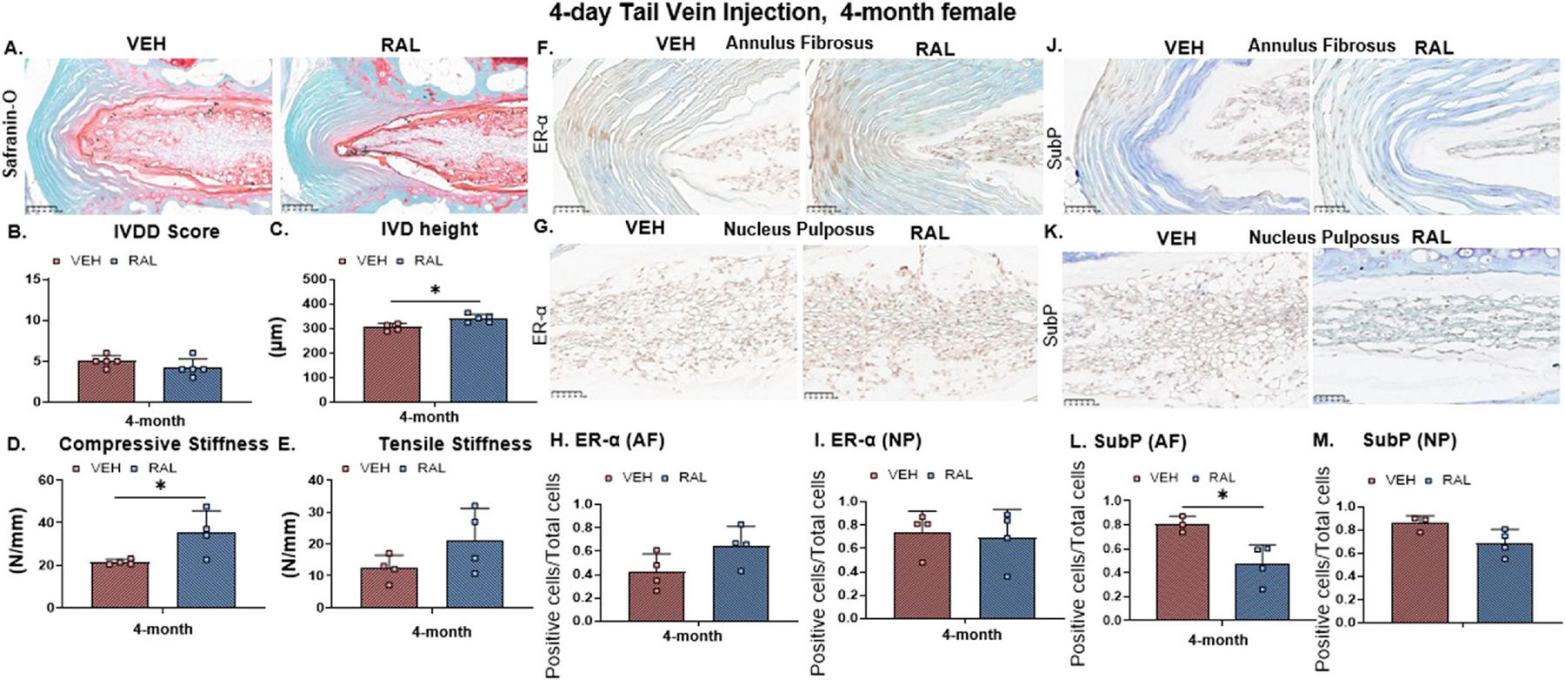
Short-term raloxifene injections enhanced IVD structure and function, and suppressed pain-related marker substance P. (A) Representative 20× histological images of 4-month-old female IVDs following 4 days of TV injection of PBS (VEH) or raloxifene (RAL). Quantification of (B) IVDD score, (C) IVD height, (D) IVD compressive stiffness and (E) IVD tensile stiffness. Representative 40× immunohistochemical images of ER-α in the (F) annulus fibrosus (AF) and (G) nucleus pulposus (NP) and quantification of the fraction of AF cells expressing estrogen receptor- α (ER-α) in the (H) AF and (I) NP. Representative 40× immunohistochemical images of substance P in the (J) AF and (K) NP and quantification of the fraction of AF cells expressing substance P in the (L) AF and (M) NP. Unpaired Student’s T-tests compared the effect of raloxifene injections (VEH vs RAL). The significance threshold for all statistical analyses was *p < 0.05. VEH-Vehicle group injected with PBS, RAL-Raloxifene injected group.

To determine the efficacy of a substantially reduced dosage of raloxifene to augment IVD structure/function/hydration, we injected the most responsive mice of 4-month-old female mice for 4 days. Although raloxifene injections did not alter the IVDD score, raloxifene injections increased IVD height by 11% (VEH:304.5/14.8, RAL:340.9/16.9) and axial compressive stiffness by 65% (VEH:21.4/1.3, RAL:35.8/12.5) without alterations to tensile stiffness nor torsional mechanical properties (Fig. 7A-E, Supplemental Figure 4A, B, Supplemental Table 8). Cellularly, short-term raloxifene injections did not alter the fraction of ER-α-positive AF cells (p=0.09), yet raloxifene injection reduced the fraction of substance P-expressing AF cells by 41% (VEH:0.8/0.1, RAL:0.5/0.2, Fig. 7F-H, J-L). Corroboratively, 4 days of raloxifene injections did not alter the gene expression of *er-α* or aggrecan (Supplemental Figure 4C, D). No changes were noted in the NP cells (Fig. 7I, M).

**Fig. 8:**
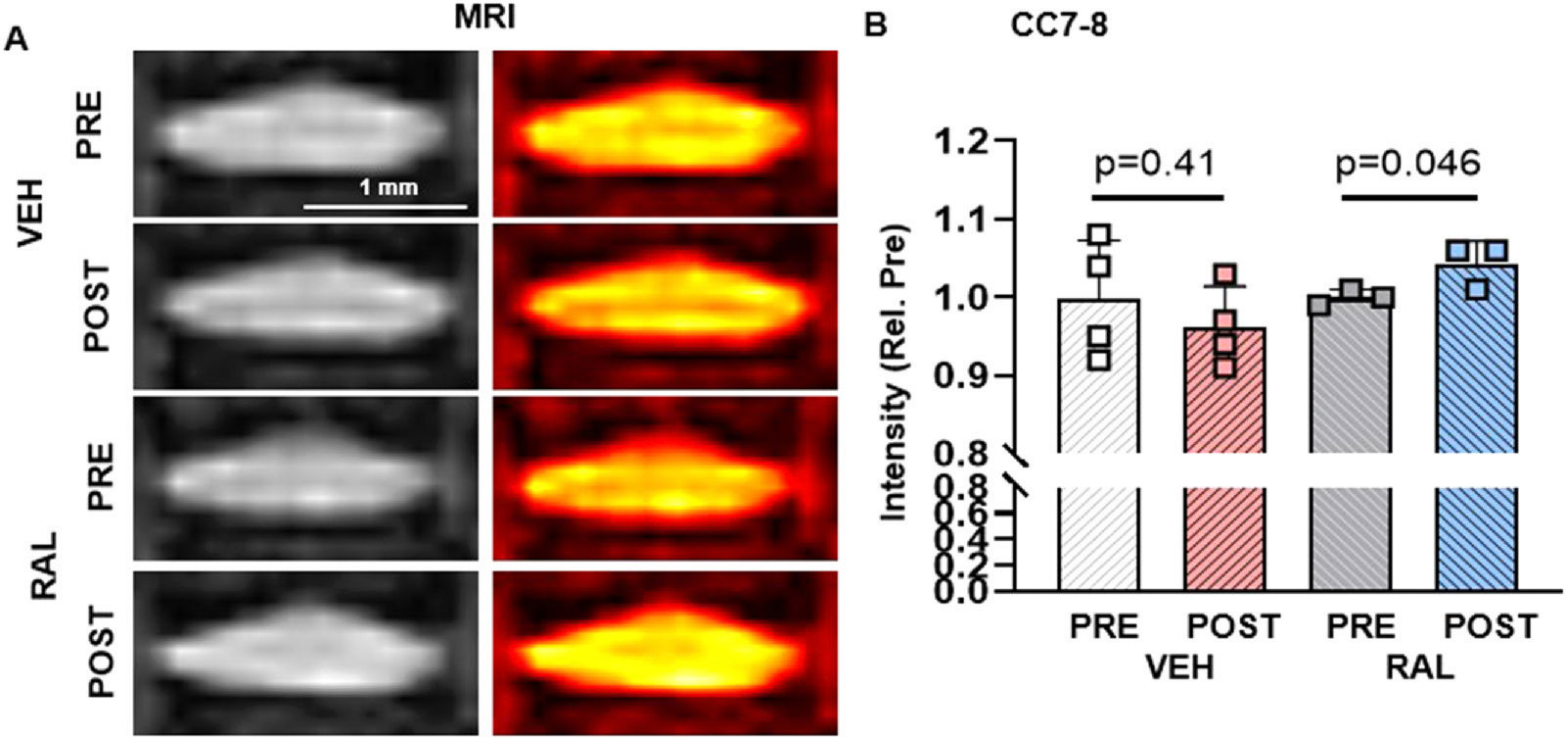
Short-term raloxifene injections for 4 days increased IVD hydration in young-adult female mice. (A left) microMRI images of CC7–8 IVDs before and after tail vein injections of PBS (VEH) or raloxifene (RAL) in 4-month-old female mice. (A right) ‘Hot’ color scheme of IVD intensity (Low-Med-High is Black-red-yellow). (B) Quantification of MRI intensity of CC7–8 IVD. Paired Student’s T-tests compared the effect of pre- vs post- VEH and RAL groups. VEH-Vehicle group injected with PBS, RAL-Raloxifene injected group. Data is normalized to pre-injection PBS (VEH) (Rel. Pre). Scale bar:1 mm.

To assess IVD hydration in 4-month-old female mice following four days of raloxifene tail vein injections, we performed microMRI on the caudal coccygeal IVDs. While MRI intensity of VEH-treated IVDs remained unchanged after injection (PRE-VEH:1/0.08, POST-VEH: 0.96/0.05), 4 daily injections of raloxifene increased MRI intensity of CC7–8, which was in the isocenter of the MRI magnet, by 4% (PRE-RAL:1/0.01, POST-RAL:1.04/0.03), indicating enhanced IVD hydration (Fig. 8A left, B). Further, altering the color scale qualitatively highlighted that raloxifene reduced the distinction between the NP cell band and the IVD (Fig. 8A right). No changes were observed in other IVDs, which were not in the isocenter of the magnet (Supplemental Figure 5A-D).

### Raloxifene injections for 4 weeks increased coccygeal trabecular bone structure and vBMD in female mice and not in male mice

Unlike in lumbar vertebrae, murine aging increases caudal coccygeal trabecular bone structure [36]. Similarly, here, aging increased caudal coccygeal trabecular and cortical bone structure and density measures in female and male mice (Supplemental Figure 6A-D; Supplemental Table 9). In female mice, raloxifene injections increased trabecular bone volume fraction (Tb.BV/TV), trabecular thickness (Tb.Th), and volumetric bone mineral density (vBMD) in 4-month-old mice by 35% (VEH: 24.33/3.23, RAL:32.92/1.20), 8% (VEH:0.06/0.00, RAL:0.07/0.00), and 31% (VEH:210.78/36.45, RAL: 276.99/43.15), respectively (Supplemental Figure 6B-D). In 24-month-old female mice, raloxifene injections trended to increase Tb.BV/TV (26%, VEH:35.18/2.51, RAL:44.34/8.42, p=0.07) and increased Tb.Th by 17% (VEH:0.07/0.00, RAL: 0.09/0.01) (Supplemental Figure 6B, C). By contrast, raloxifene injections did not alter trabecular bone structure or vBMD in males.

## Discussion

Raloxifene injections augment IVD structure and function, and reduce the number of IVD cells stained positively for neurotransmitter substance P in young-adult female and male mice [22,23], yet further studies are needed to determine whether raloxifene injections would (i) improve evoked and spontaneous pain-related behavior, (ii) whether aging would attenuate the IVD structural benefits of raloxifene injections in female and male mice, and (iii) whether short-term injections of raloxifene could improve IVD structure, function and hydration despite unlikely stimulation of ER signaling. Here, raloxifene injections reduced the number of IVD cells expressing substance P and reduced the evoked response to mechanical stimuli in young-adult and old, female and male mice. Distinct by sex, raloxifene injections reduced IVDD grade, promoted extracellular matrix constituents, increased biomechanical compressive stiffness, and trended to increase bound water in young-adult female mice, but raloxifene injections did not promote these changes in young-adult male mice. In females, the benefits of raloxifene injections to IVD structure persisted in old mice and a lower dose of raloxifene injections promoted IVD hydration. Further, raloxifene injections in old female mice promoted the percentage of the stance spent propelling rather than braking, suggesting more effective forward motion. Our findings suggest that raloxifene injections promote IVD structure and attenuate age-related evoked and spontaneous pain behavior in female mice with fewer benefits in male mice.

Similar to our previous study [22], raloxifene injections increased the number of ERα-positive IVD cells, improved IVD and bone structure, increased extracellular matrix constituents and subsequently stiffened IVD in young-adult female mice with limited age-related impairment in old female mice, but with sex-related impairment in the response of young-adult male mice. Although raloxifene injections rescued the age-related decline in the number ERα-positive IVD cells of female and male mice, the sex-related impairment of young-adult male IVD and bone structure to raloxifene persisted in old male mice and the improvement in gait dynamics in old female mice did not carry over to old male mice. Clinically, raloxifene (Evista) is approved for osteoporosis treatment in postmenopausal women, may increase IVD height [20,22], reduces self-reported back pain [37] and acts as a non-uterine estrogen agonist in bone and IVD tissues [38]. By contrast, raloxifene is not currently prescribed to men, but the impaired accrual of vertebral bone and promotion of IVD structure in male mice may be a species-dependent phenomenon [22,39]. Serum levels of estradiol are associated with vertebral bone mineral density in older men [40] and raloxifene increases estradiol and bone mineral density in healthy middle-aged men with low sex hormones and in men undergoing gonadal suppression therapy [41,42].

Despite limited benefits of raloxifene injections to IVD structure in young-adult or old male mice, raloxifene injections reduced the force required to evoke a response by mechanical stimuli in female and male, young-adult and old mice. The literature supports the concept that pain is not entirely discogenic. For instance, cell transplantation clinical trials for discogenic back pain show improvements in pain with lesser evidence for IVD repair or regeneration [43]. Similarly, patients with chronic low back pain may experience symptom relief following intradiscal methylene blue injection despite minimal changes in IVD height on imaging [44]. Likewise, in rodent models, dietary polyphenols can alleviate IVD injury-induced pain without altering IVD height [45]. In contrast, clinical studies show that even IVDs with an intact annulus fibrosus can be significant sources of pain, potentially due to central sensitization or neuroinflammatory processes [46,47], highlighting that discogenic pain mechanisms are distinct from structural and mechanical IVDD, even though they may associate.

Lastly, the benefits of raloxifene to IVD and bone structure must be balanced with its iatrogenic effects. While low-to-high doses of raloxifene (e.g., 30 mg vs 60 mg vs 150 mg daily) in early postmenopausal women reduce bone turnover markers (1–2%), moderate-to-high doses also significantly increase adverse effects such as hot flashes (47%) and breast pain (57%) over prolonged use of 36 months [48]. In our study, short-term (4 days) injections of raloxifene improved IVD height, biomechanical stiffness and IVD hydration without significant IVD estrogen signaling, suggesting that the biomechanical benefits from injections of raloxifene may have estrogen independent mechanisms rather than by the slow synthesis rates of proteoglycan by IVD cells [49]. An analog of raloxifene that reduces the binding of raloxifene to estrogen receptors, yet retains its affinity to collagen-binding, promotes bone structure and mechanical toughness [50,51]. Therefore, this raloxifene analog may also induce similar functional IVD benefits to females and males by increasing the hydration, while potentially minimizing the estrogenic side effects [41,42].

These data suggested that raloxifene injections augment IVD structure and mitigate pain-related behavior in old mice, which may have translational implications, despite limited attenuation of age-related spontaneous measures of altered gait in male mice. Pre-clinical and experimental design limitations of the study may have precluded the interpretation of the potential response in men. Next, the sensitivity of the response of male mice to raloxifene may be species-dependent and not model the potential response in men [22,39]. Further, few comparisons were underpowered based on our initial power analysis of ER-α protein upregulation in IVD cells [22]. The sample size calculations were conducted for the response in female mice, which may have underpowered the sample size number necessary for outcomes in male mice to reach statistical significance. Further, the statistical approach did not directly compare sex effects from injections of raloxifene, but raloxifene injections in male mice was compared to vehicle-treated mice and corroborated minimal effects. Lastly, we excluded samples that failed to meet our quality control standards or encountered technical issues.

Overall, raloxifene injections promoted IVD structure and reduced pain-related behavior in young-adult and old mice, indicating that aging did not impair the response to raloxifene. Raloxifene injections reduced the sensitivity to mechanical stimuli and the neuropeptide substance P within the IVD in young-adult and old, female and male mice. Further, a lower dose of raloxifene than normally prescribed may retain its benefits to spinal structure and pain-reduction while also reducing the side effects noted in the literature, but this requires further investigation. This study contributes to the growing understanding of the skeletal benefits and pain relief by raloxifene and demonstrates the potential for raloxifene to treat IVDD and/or pain in women and men.

## Supplementary Material

MMC3

MMC1

MMC6

MMC7

MMC11

MMC14

MMC9

MMC13

MMC2

MMC8

MMC5

MMC10

MMC4

MMC12

## Data Availability

All data are available in the main text or the supplementary material.

## Appendix A. Supporting information

Supplementary data associated with this article can be found in the online version at doi:10.1016/j.joca.2026.03.118.
