# Supplementary material for: Raloxifene injections normalize age-related mechanical sensitization in female and male mice and augment intervertebral disc structure in old female mice
Source: Osteoarthritis Cartilage. Author manuscript; Available in PMC 2026 Jun 3. (PMC13228093; doi:10.1016/j.joca.2026.03.118)
Supplement: MMC3 [file NIHMS2166731-supplement-MMC3.docx]

| **Figure** | **Group**  **Supplemental Table 3: Main Spinal Level Outcomes with Sample size and Exclusion Criteria** | **Sample Size (n)** | **Notes/Exclusions** |
| --- | --- | --- | --- |
| **Fig.1 1A.** | 4 mo Female | VEH = 5, Ral = 5 | N/A |
|  | 24 mo Female | VEH = 4, Ral = 5 | N/A |
|  | 4 mo Male | VEH = 5, Ral = 5 | N/A |
|  | 24 mo Male | VEH = 4, Ral = 4 | N/A |
| **1B.** | 4 mo Female | VEH = 5, Ral = 5 | N/A |
|  | 24 mo Female | VEH = 3, Ral = 5 | One aged female unwilling to walk |
|  | 4 mo Male | VEH = 5, Ral = 4 | N/A |
|  | 24 mo Male | VEH = 4, Ral = 5 | N/A |
| **Fig. 2 2B.** | 4 mo Female | VEH = 3, Ral = 5 | Extreme histological artifacts |
|  | 24 mo Female | VEH = 4, Ral = 5 | N/A |
|  | 4 mo Male | VEH = 4, Ral = 3 | Extreme histological artifacts |
|  | 24 mo Male | VEH = 4, Ral = 5 | N/A |
| **2C.** | 4 mo Female | VEH = 3, Ral = 3 | Extreme histological artifacts |
|  | 24 mo Female | VEH = 4, Ral = 5 | N/A |
|  | 4 mo Male | VEH = 4, Ral = 3 | Extreme histological artifacts |
|  | 24 mo Male | VEH = 4, Ral = 5 | N/A |
| **Fig. 3 3B.** | 4 mo Female | VEH = 5, Ral = 3 | Extreme histological artifacts |
|  | 24 mo Female | VEH = 3, Ral = 4 | N/A |
|  | 4 mo Male | VEH = 4, Ral = 3 | Extreme histological artifacts |
|  | 24 mo Male | VEH = 4, Ral = 4 | N/A |
| **3C.** | 4 mo Female | VEH = 5, Ral = 3 | Extreme histological artifacts |
|  | 24 mo Female | VEH = 3, Ral = 4 | Extreme histological artifacts |
|  | 4 mo Male | VEH = 4, Ral = 3 | Extreme histological artifacts |
|  | 24 mo Male | VEH = 4, Ral = 4 | N/A |
| **Fig. 4 4E.** | 4 mo Female | VEH = 4, Ral = 4 | N/A |
|  | 24 mo Female | VEH = 5, Ral = 5 | N/A |
|  | 4 mo Male | VEH = 5, Ral = 5 | N/A |
|  | 24 mo Male | VEH = 4, Ral = 4 | N/A |
| **4F.** | Same as 4E | Same as 4E | N/A |
| **Fig. 5 5B.** | 4 mo Female | VEH = 5, Ral = 5 | N/A |
|  | 24 mo Female | VEH = 5, Ral = 5 | N/A |
|  | 4 mo Male | VEH = 4, Ral = 4 | N/A |
|  | 24 mo Male | VEH = 4, Ral = 5 | N/A |
| **5C.** | Same as 5B | Same as 5B | N/A |
| **5D.** | Same as 5B | Same as 5B | N/A |
| **Fig. 6 6A.** | 4 mo Female | VEH = 4, Ral = 5 | N/A |
|  | 24 mo Female | VEH = 5, Ral = 4 | N/A |
|  | 4 mo Male | VEH = 5, Ral = 5 | N/A |
|  | 24 mo Male | VEH = 4, Ral = 4 | N/A |
| **6B.** | Same as 6A | Same as 6A | N/A |
| **Fig. 7 7B.** | 4 mo Female | VEH = 5, Ral = 5 | N/A |
| **7C.** | 4 mo Female | VEH = 4, Ral = 5 | N/A |
| **7D.** | 4 mo Female | VEH = 4, Ral = 4 | N/A |
| **7E.** | 4 mo Female | VEH = 4, Ral = 4 | N/A |
| **7H-I.** | 4 mo Female | VEH = 4, Ral = 4 | N/A |
| **7L-M.** | 4 mo Female | VEH = 3, Ral = 4 | Extreme histological artifacts |
| **Fig. 8 8B** | 4 mo Female | VEH = 4 pre / 4 post | N/A |
|  |  | Ral = 3 pre / 3 post | *Premature death from low body mass* |
