# Supplementary figures and images for: Raloxifene injections normalize age-related mechanical sensitization in female and male mice and augment intervertebral disc structure in old female mice
Source: Osteoarthritis Cartilage. Author manuscript; Available in PMC 2026 Jun 3. (PMC13228093; doi:10.1016/j.joca.2026.03.118)

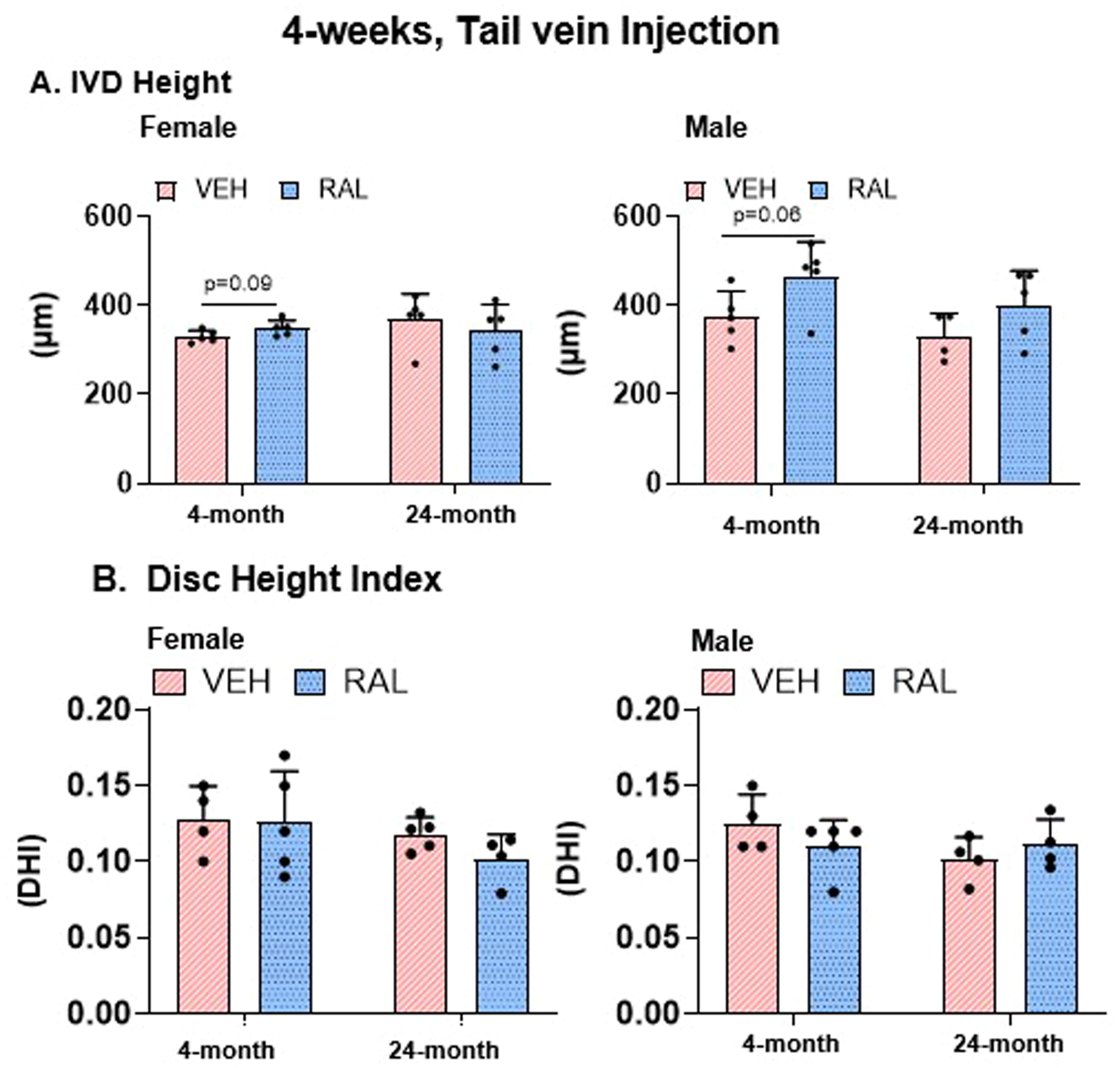

Supplement: MMC11 [file NIHMS2166731-supplement-MMC11.jpg]

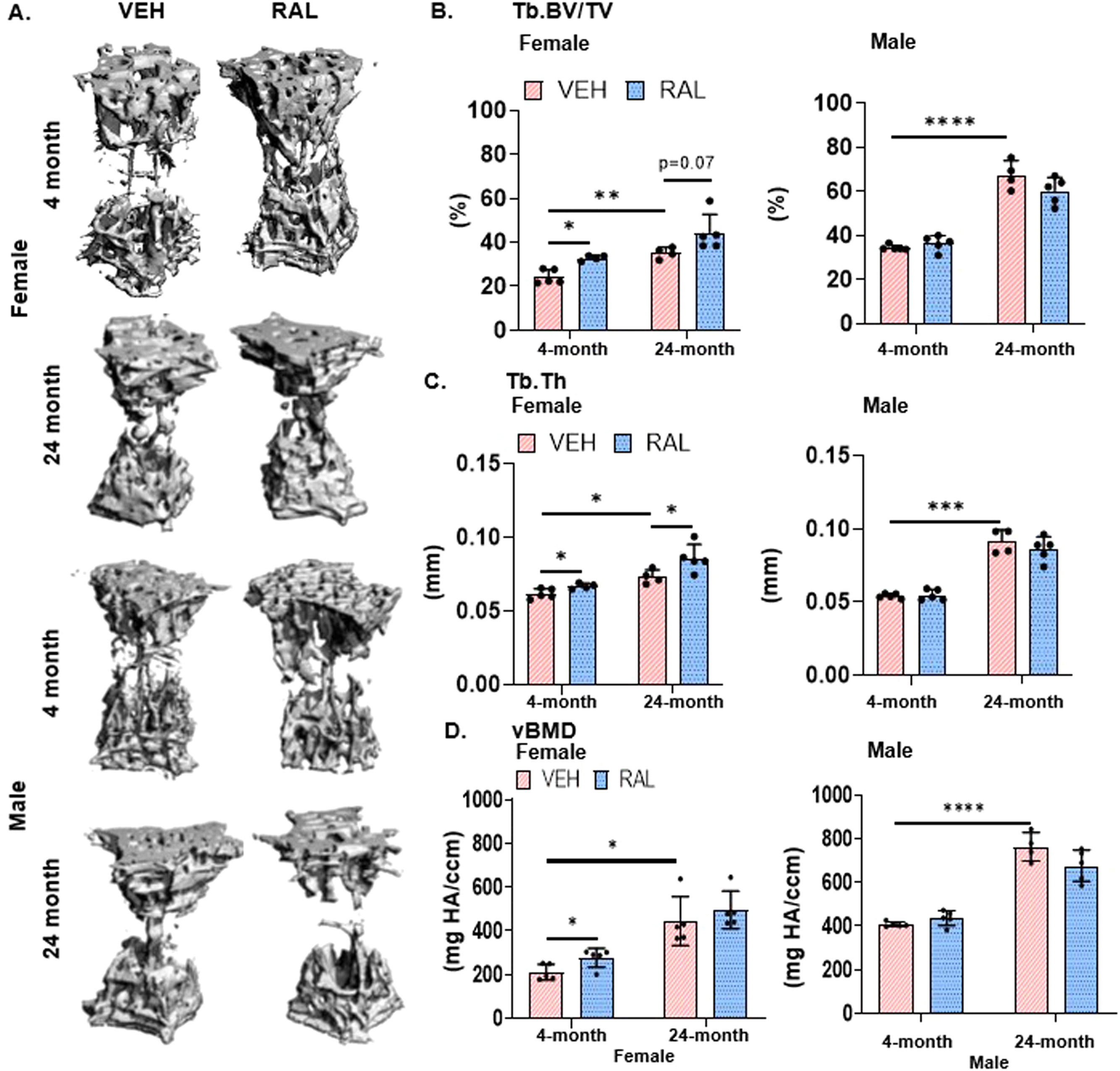

Supplement: MMC14 [file NIHMS2166731-supplement-MMC14.jpg]

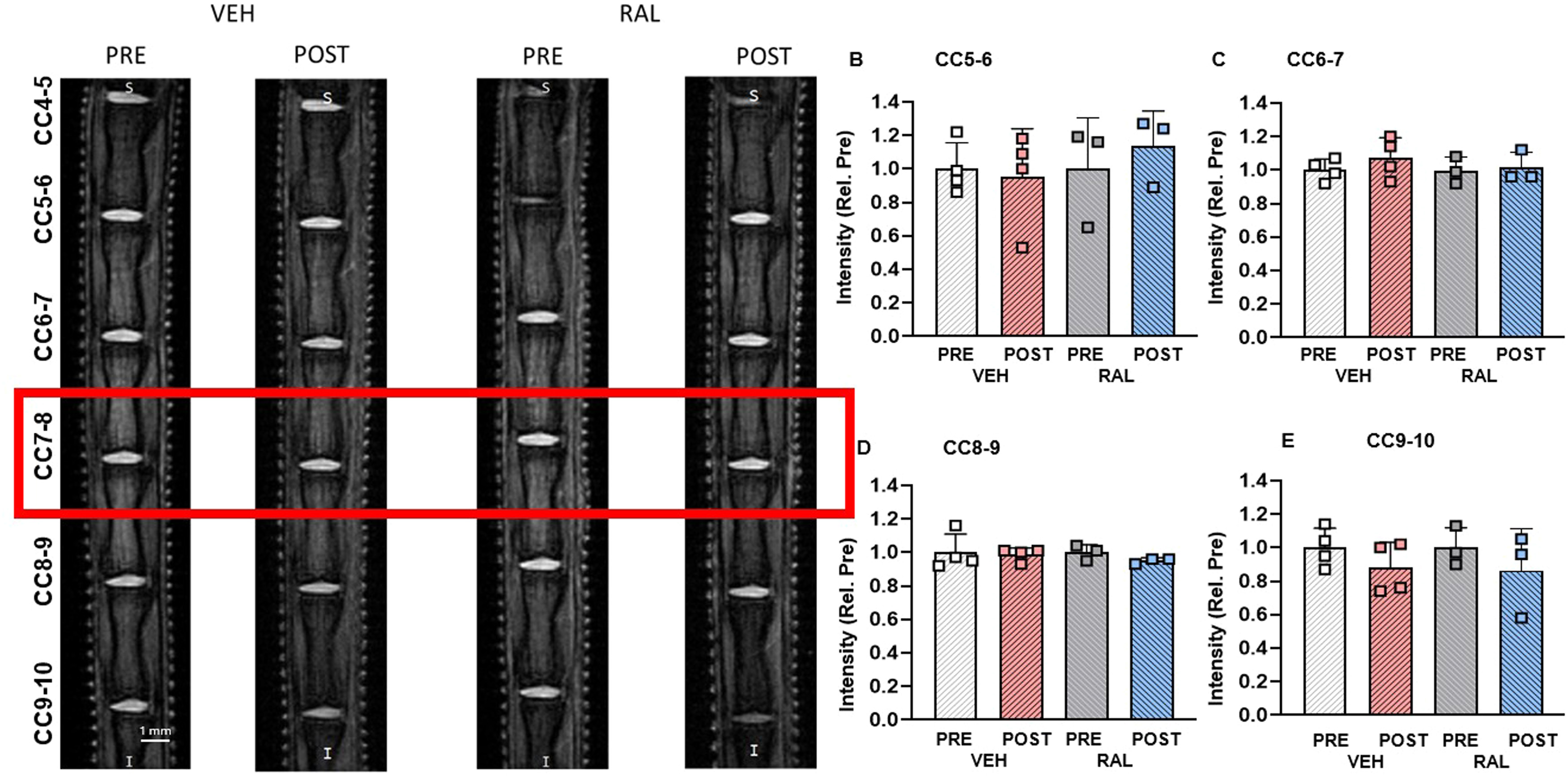

Supplement: MMC13 [file NIHMS2166731-supplement-MMC13.jpg]

**Supplemental Table 2: Spinal Level Outcomes**


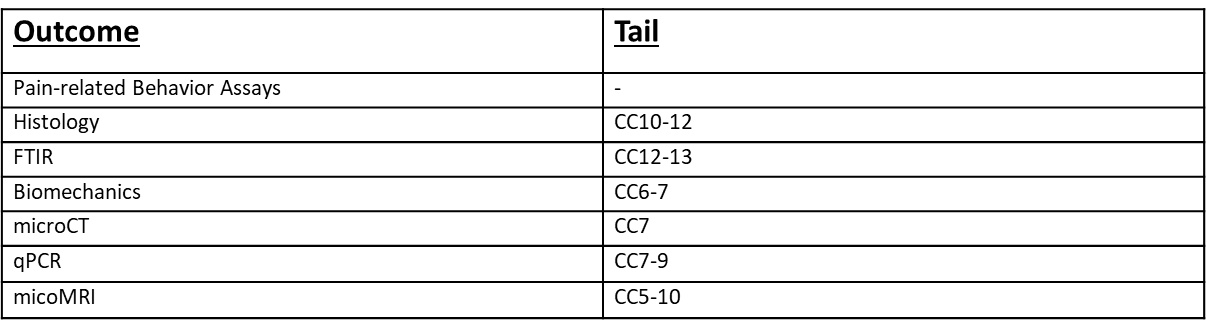

Supplement: MMC2 [file NIHMS2166731-supplement-MMC2.docx]

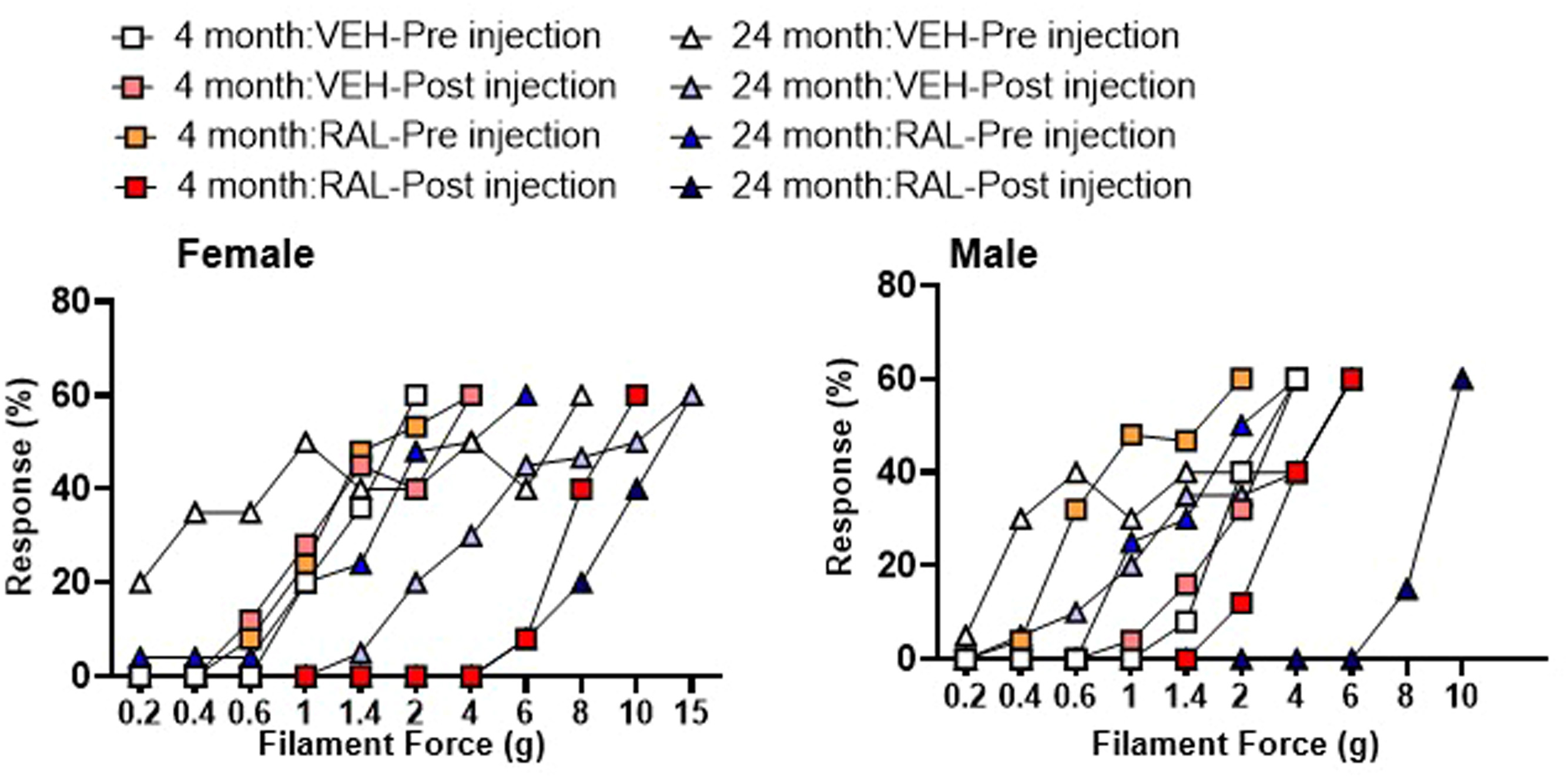

Supplement: MMC10 [file NIHMS2166731-supplement-MMC10.jpg]

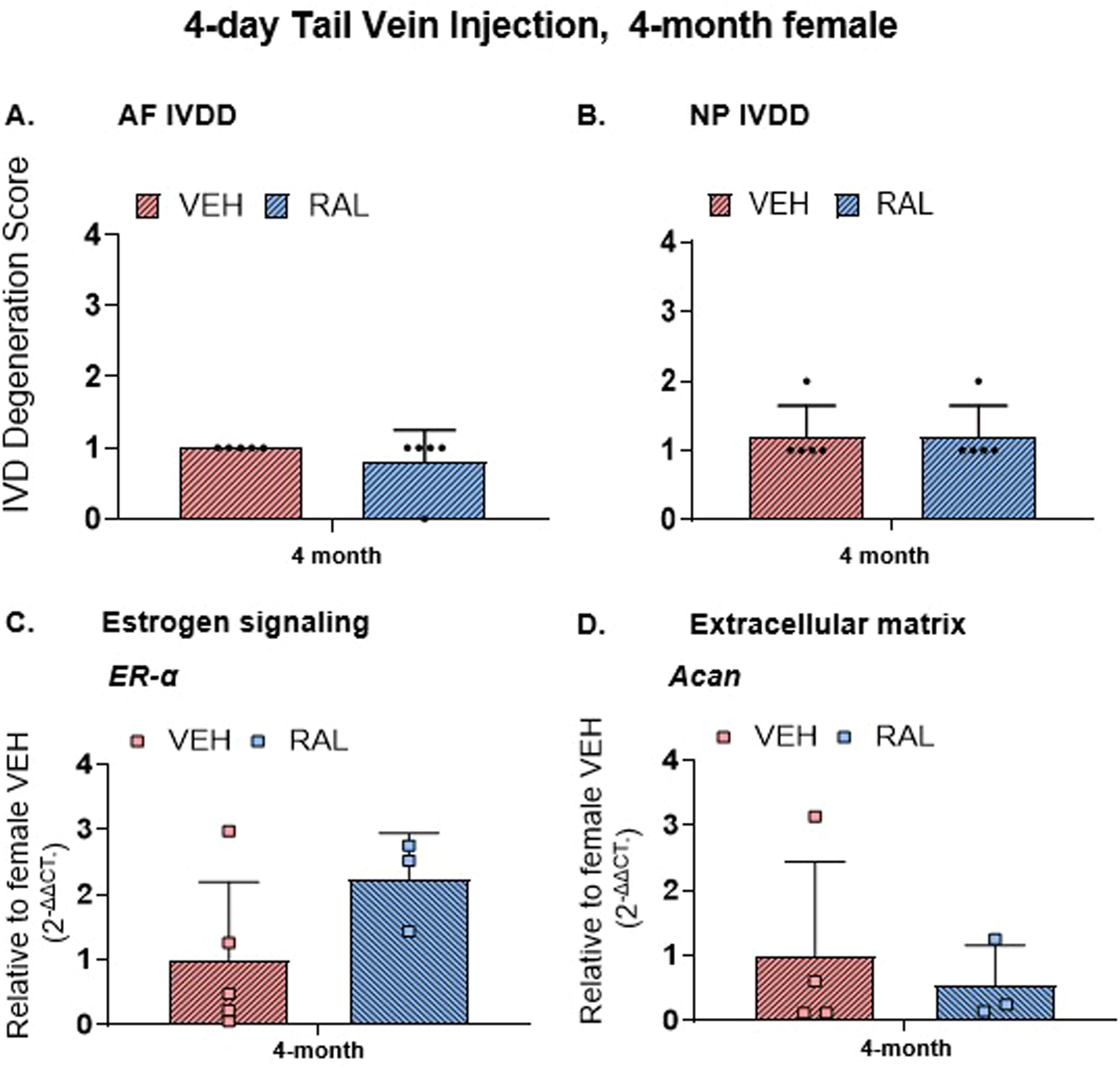

Supplement: MMC12 [file NIHMS2166731-supplement-MMC12.jpg]
