# Supplementary material for: Raloxifene injections normalize age-related mechanical sensitization in female and male mice and augment intervertebral disc structure in old female mice
Source: Osteoarthritis Cartilage. Author manuscript; Available in PMC 2026 Jun 3. (PMC13228093; doi:10.1016/j.joca.2026.03.118)
Supplement: MMC9 [file NIHMS2166731-supplement-MMC9.docx]

**Supplemental Table 9: Trabecular and Cortical vertebral bone analysis for pre and post tail Intravenous injection of vehicle or raloxifene in young-adult and old, female and male mice**


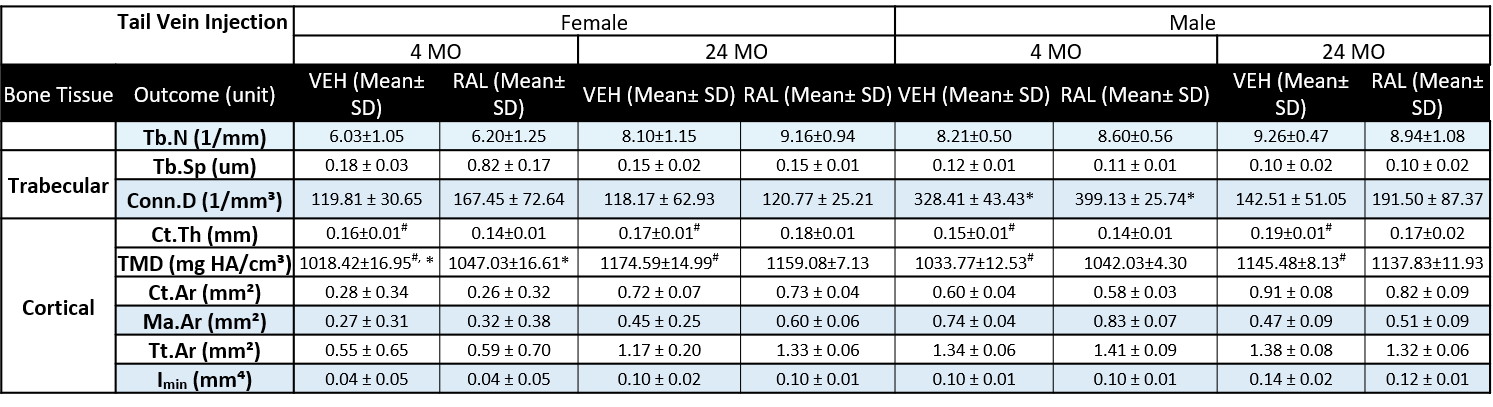


^#^ 4- vs 24- mo VEH

* VEH vs RAL
