# Supplementary material for: Raloxifene injections normalize age-related mechanical sensitization in female and male mice and augment intervertebral disc structure in old female mice
Source: Osteoarthritis Cartilage. Author manuscript; Available in PMC 2026 Jun 3. (PMC13228093; doi:10.1016/j.joca.2026.03.118)

**Supplemental Table 5: Gait analysis for pre and post tail vein intravenous injection of vehicle or raloxifene in young-adult and old, female and male mice**

*pre- vs post- injection


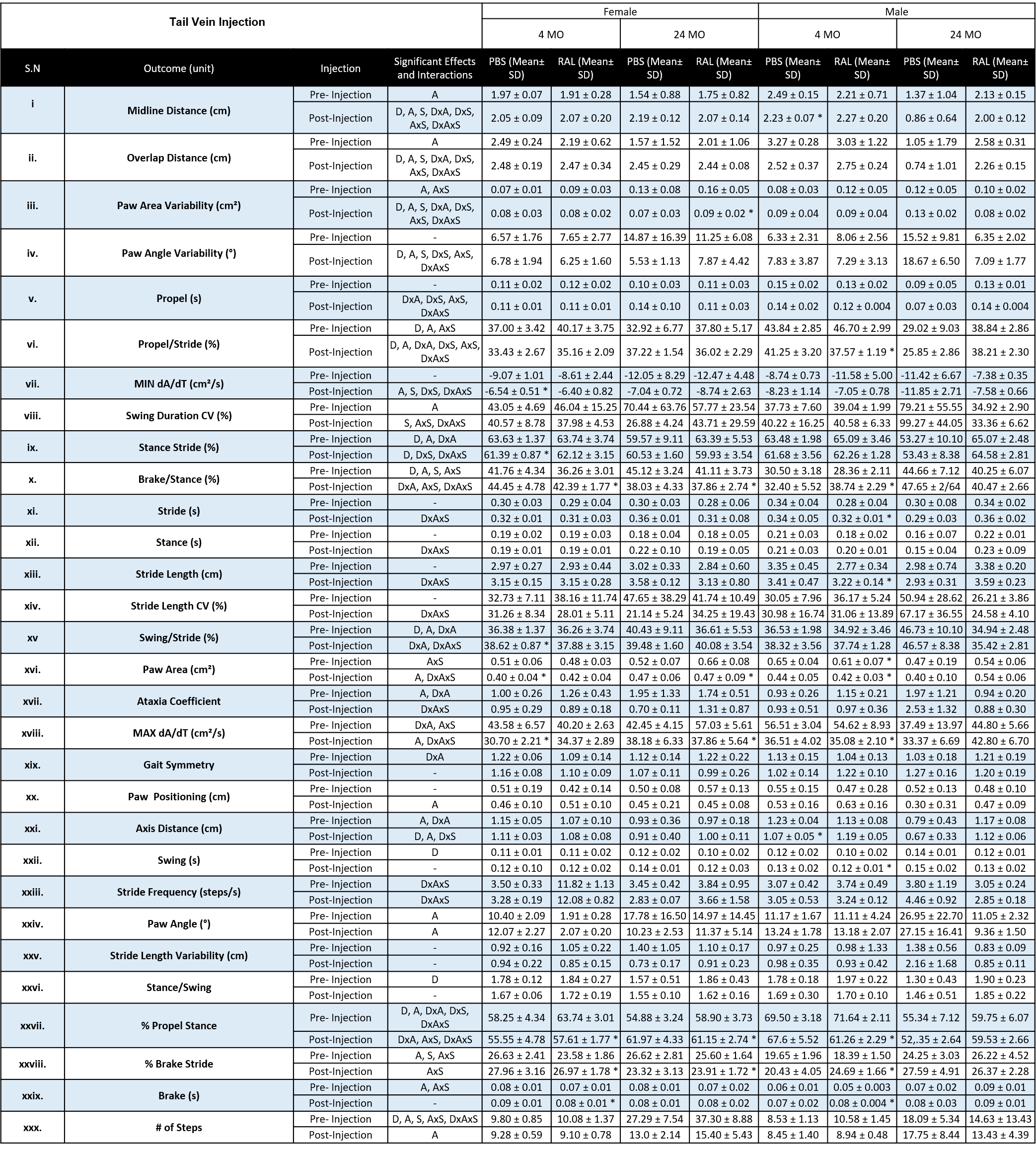

Supplement: MMC5 [file NIHMS2166731-supplement-MMC5.docx]
