# Supplementary material for: Raloxifene injections normalize age-related mechanical sensitization in female and male mice and augment intervertebral disc structure in old female mice
Source: Osteoarthritis Cartilage. Author manuscript; Available in PMC 2026 Jun 3. (PMC13228093; doi:10.1016/j.joca.2026.03.118)
Supplement: MMC4 [file NIHMS2166731-supplement-MMC4.docx]

**Supplemental Table 4: Supplemental Spinal Level Outcomes with Sample size and Exclusion Criteria**

| **Figure** | **Group** | **Sample Size (n)** | **Notes/Exclusions** |
| --- | --- | --- | --- |
| **Sup Fig. 1 1B.** | 4 mo Female | VEH = 5, Ral = 8 | N/A |
|  | 24 mo Female | VEH = 5, Ral = 5 | N/A |
| **1C.** | 4 mo Female | VEH = 6, Ral = 6 | N/A |
|  | 24 mo Female | VEH = 5, Ral = 5 | N/A |
| **1D.** | 4 mo Female | VEH = 8, Ral = 5 | N/A |
|  | 24 mo Female | VEH = 8, Ral = 8 | N/A |
| **1E.** | 4 mo Female | VEH = 7, Ral = 5 | N/A |
|  | 24 mo Female | VEH = 8, Ral = 8 | N/A |
| **Sup Fig. 2 2A.** | 4 mo Female | VEH = 5, Ral = 5 | N/A |
|  | 24 mo Female | VEH = 4, Ral = 5 | N/A |
|  | 4 mo Male | VEH = 5, Ral = 5 | N/A |
|  | 24 mo Male | VEH = 4, Ral = 4 | N/A |
| **Sup Fig. 3 3A.** | 4 mo Female | VEH = 5, Ral = 5 | N/A |
|  | 24 mo Female | VEH = 5, Ral = 5 | N/A |
|  | 4 mo Male | VEH = 5, Ral = 5 | N/A |
|  | 24 mo Male | VEH = 4, Ral = 5 | N/A |
| **3B.** | 4 mo Female | VEH = 4, Ral = 5 | N/A |
|  | 24 mo Female | VEH = 5, Ral = 4 | N/A |
|  | 4 mo Male | VEH = 5, Ral = 5 | N/A |
|  | 24 mo Male | VEH = 4, Ral = 4 | N/A |
| **Sup Fig. 4 4A.**  **4B.** | 4 mo Female | VEH = 5, Ral = 5 | N/A |
| **4C.**  **4D.** | 4 mo Female | VEH = 4, Ral = 3 | QPCR exclusion: poor mRNA quality |
| **Sup Fig. 5 5A-D.** | 4 mo Female | VEH = 4 pre / 4 post | N/A |
|  |  | Ral = 3 pre / 3 post | *Premature death from low body mass* |
| **Sup Fig. 6** | 4 mo Female | VEH = 5, Ral = 4 | N/A |
|  | 24 mo Female | VEH = 4, Ral = 5 | N/A |
|  | 4 mo Male | VEH = 5, Ral = 5 | N/A |
|  | 24 mo Male | VEH = 4, Ral = 5 | N/A |
